# HuD Promotes BDNF Expression in Brain Neurons via Selective Stabilization of the BDNF Long 3′UTR mRNA

**DOI:** 10.1371/journal.pone.0055718

**Published:** 2013-01-31

**Authors:** Megan Allen, Clark Bird, Wei Feng, Guanglu Liu, Wenqi Li, Nora I. Perrone-Bizzozero, Yue Feng

**Affiliations:** 1 Department of Pharmacology, Emory University School of Medicine, Atlanta, Georgia, United States of America; 2 Department of Neurosciences, University of New Mexico School of Medicine, Albuquerque, New Mexico, United States of America; University of Louisville, United States of America

## Abstract

Complex regulation of brain-derived neurotrophic factor (BDNF) governs its intricate functions in brain development and neuronal plasticity. Besides tight transcriptional control from multiple distinct promoters, alternative 3′end processing of the BDNF transcripts generates either a long or a short 3′untranslated region (3′UTR). Previous reports indicate that distinct RNA sequence in the BDNF 3′UTRs differentially regulates BDNF production in the brain to accommodate neuronal activity changes, conceivably through differential interactions with undefined trans-acting factors that regulate stability and translation of these BDNF mRNA isoforms. In this study, we report that the neuronal RNA-binding protein (RBP) HuD interacts with a highly conserved AU-rich element (ARE) specifically located in the BDNF long 3′UTR. Such interaction is necessary and sufficient for selective stabilization of mRNAs that contain the BDNF long 3′UTR *in vitro* and *in vivo*. Moreover, in a HuD transgenic mouse model, the BDNF long 3′UTR mRNA is increased in the hippocampal dentate granule cells (DGCs), leading to elevated expression of BDNF protein that is transported and stored in the mossy fiber (MF) terminals. Our results identify HuD as the first trans-acting factor that enhances BDNF expression specifically through the long 3′UTR and a novel mechanism that regulates BDNF protein production in selected neuronal populations by HuD abundance.

## Introduction

Brain-derived neurotrophic factor (BDNF) plays pivotal roles in governing a broad spectrum of brain functions including neuronal survival, neural network development, and synaptic plasticity. To accommodate such intricate functions, BDNF expression is under precise regulation. Furthermore, dysregulation of BDNF is indicated in the pathogenesis of various neurological and psychiatric diseases [1], [2]. Transcription of BDNF can be initiated from at least eight different promoters in mammals [3], [4], which underlies distinct responses to various stimulation cues [3], [5], [6]. Besides the sophisticated transcriptional regulation, alternative polyadenylation of the BDNF transcripts results in two pools of BDNF mRNAs that carry either a short or a long 3′untranslated region (UTR), regardless of which promoter drives BDNF transcription [3], [7]. While the entire sequence of the short 3′UTR is contained within the long 3′UTR, it is the unique sequence in the BDNF long 3′ UTR that mediates differential regulation of BDNF production through multiple posttranscriptional mechanisms. For instance, the long 3′UTR, but not the short 3′UTR, suppresses BDNF translation in the resting brain and mediates neuronal activity-dependent translation of BDNF [8]. The long 3′UTR can also target BDNF mRNA into dendrites thereby governing dendritic BDNF protein abundance [8], [9]. Furthermore, the short and long 3′UTR differentially affect BDNF mRNA stability, with the long transcript having a much shorter half-life than its short counterpart [10]. Mechanistically, the unique sequence in the BDNF long 3′UTR provides binding sites for trans-acting RNA-binding proteins (RBPs) and/or microRNAs to achieve differential posttranscriptional regulation of BDNF mRNA isoforms [11]. However, no previous studies have identified any RBPs that specifically bind and regulate the BDNF long 3′UTR mRNA in brain neurons.

We report here that HuD, a neuron-specific RBP that plays critical roles in governing neuronal circuitry development and plasticity via controlling mRNA stability and/or translation [12], selectively binds the BDNF long 3′UTR but not the short 3′UTR. A highly conserved AU-rich element (ARE), located at the distal end of the BDNF long 3′UTR, mediates direct interaction with HuD in vitro and in transfected cells, which is necessary for HuD to stabilize reporter RNAs that harbor the BDNF long 3′UTR. Moreover, shRNA-mediated HuD knockdown results in overall reduction of the BDNF long 3′UTR mRNA in the soma and processes of hippocampal neurons whereas over-expression of HuD leads to selectively enhanced expression of the BDNF long 3′UTR mRNA. Finally, we show that over-expression of HuD from a transgene increases BDNF long 3′UTR mRNA levels primarily in the hippocampal dentate granule cells (DGCs) and BDNF protein accumulation in the hippocampal mossy fiber (MFs) axon terminals projected from DGCs. Together, our findings identify a novel mechanism for posttranscriptional regulation of distinct BDNF mRNA isoforms, which reveals a functional link between the pathways under HuD and BDNF, both playing key roles in controlling plasticity of neuronal circuitry.

## Materials and Methods

### Cell culture, plasmids and transfection

The ARE at the distal end of BDNF long 3′UTR was removed by PCR amplification of the pcLuc-BDNFlongUTR plasmid [8] with primers of ccgctcgagTGGATTTATGTTGTATAGATTAT (forward) and gctctagaACATGGTGAATAATATCTTTACC (reverse), and subcloned between the XhoI/XbaI sites to replace the full length BDNF 3′UTR in pcLuc-BDNF longUTR (pcLuc-BDNF longUTRΔARE). Luciferase reporter constructs were either transfected alone, co-transfected with myc-HuD [13] or pcDNA into the immortalized mouse brain neuron cell line CAD using Lipofectamine 2000 (Invitrogen) as indicated in the corresponding figure legends. The pRL-TK renilla luciferase construct was co-transfected for assessing transfection efficiency in luciferase assay. Reporter expression was quantified using the Dual-Luciferase assay (Promega).

### UV-crosslinking-immunoprecipitation (CLIP) and RT-PCR

UV-crosslinking was performed as previously described [14] with modifications. Briefly, cells were exposed to UV light in a Stratalinker (Stratagene, 400 mJ). Cells were then lysed in ice-cold buffer containing 25 mM Tris, 150 mM NaCl, 0.5% Triton X-100, and protease inhibitors. The postnuclear supernatant was precleared with IgG-conjugated protein A-Sepharose beads in the presence of 0.001% SDS followed by immunoprecipitation using anti-c-myc antibody (1∶500, SantaCruz) conjugated to protein A beads as described in our previous study [15]. The immunoprecipitated complexes were proteinase K-treated before RNA extraction [16]. RT-PCR was performed with Primer A: ATCAGGCAAGGATATGGGCTCACT (forward) and TCCAGATCCACAACCTTCGCTTCA (reverse); Primer B: TGGCCTAACAGTGTTTGCAG (forward) and GGATTTGAGTGTGGTTCTCC (reverse); and Primer C: CAGTGGCTGGCTCTCTTACC (forward) and GGCCACAGACATTTACTTACAGTTT (reverse).

### Actinomycin D treatment and reporter mRNA decay in transfectected cells

The BDNF-L-3′UTR plasmid was transfected into CAD cells with either the myc-HuD construct or the parental vector control. 24 hours post-transfection, cells were treated with actinomycin D at the concentration of 8 µg/mL and harvested at 4, 8, and 12 hours. qRT-PCR was performed using DNase-treated total RNA isolated from each time-point using luciferase primers. Percentage of remaining reporter mRNA at each time point was calculated by normalizing the qRT-PCR reading to that of time zero and plotted against time.

### In vitro mRNA binding and decay

A 164 bp fragment containing the BDNF ARE in the long 3′UTR (nt 2640–2746) was PCR-amplified and cloned into the XbaI/XhoI sites of pBSKII (Invitrogen). Sequencing results confirmed 100% identity as compared to published sequence. Radiolabeled BDNF-ARE RNA was prepared by *in vitro* transcription using ^32^P-UTP as described [17]. RNA electrophoretic mobility shift assay [REMSA, [18]] used 100,000 cpm of ^32^P-UTP labeled BDNF-ARE RNA, increasing amounts of purified GST or GST-HuD [19], and 0.25 mg/ml yeast tRNA and 0.25 mg/ml of BSA to minimize non-specific interactions. Specific competition was carried out with a 10-fold molar excess of cold BDNF-ARE RNA. *In vitro* mRNA decay reactions were performed using ∼200 fmol (100,000 cpm) of capped and polyadenylated radiolabeled BDNF-ARE RNA and 20 µg S100 protein from HuD-KO mice. Reactions were supplemented with either 50 ng of GST-HuD or GST and the half-life of the mRNA was calculated as described [20].

### Treatment of Primary cultures of embryonic cortical and hippocampal neurons

Animal treatment was in compliance to NIH regulations under the approval of IACUC by the Emory University and University of New Mexico. Neuronal cell cultures were prepared from E17 C57BL/6 mice [21], and were grown for 24 hours before infection with HSV-HuD or control HSV-lacZ as described [22]. After 72 h, total RNA was isolated and BDNF long 3′UTR mRNA and pan BDNF mRNA were quantified by RT-qPCR using GAPDH mRNA as an internal reference.

For shRNA-mediated HuD knockdown, 100,000 E17 hippocampal neurons were grown for 4 days on poly-L lysine coated coverslips and then transfected with either pRetro-shHuD plasmid [23] and pEGFP (Clontech) or control pRetro vector and pEGFP using Lipofectamine™ 2000 (Life technologies). Following 48 h, cells were fixed in 4% PFA and prepared for FISH as described below. To knockdown HuD in CAD cells, 200 npmol of siHuD that harbors identical sequence to the targeting sequence in shHuD, or a negative control siRNA (Invitrogene), was transfected into CAD cells. Total RNA was collected 48 hrs after transfection, followed by qRT-PCR to quantify HuD and BDNF long mRNA respectively. GAPDH mRNA was used as an internal reference for normalization.

### In situ hybridization and immunofluorescence

Fluorescent in situ hybridization (FISH) was performed using a digoxigenin-labeled antisense oligonucleotide complementary to nucleotides 2508–2556 in the BDNF long 3′ UTR (5′ GGGTGTATACAATAACTTTTATCTGCAAACACGTTAGG-CCATATTAC) as described [24]. For FISH of brain slices, duplicate adjacent cryostat brain sections from HuD-Tg mice and non-transgenic wild type littermates (WT) were analyzed in parallel and images acquired using the same exposure (typically 50 ms with a 40× objective) using an Olympus BX-60 microscope with a DP71 CCD-digital camera (Olympus America). A similar protocol was used for FISH of hippocampal neurons in culture with the following modifications to detect both the signal from transfected GFP and L-BDNF mRNAs. After the hybridization with dig-labeled probes, cells were incubated with sheep anti-dig antibodies (Roche) and mouse anti-GFP antibodies (Millipore) followed by Alexa 488-conjugated anti-mouse secondary antibodies (Life Technologies) and Cy3 TSA reagent (Perkin-Elmer). Images were acquired in a Zeiss LSM 510 confocal microscope using a 63× objective and 0.8 µm optical slices.

To assess BDNF protein levels in the hippocampus, brain slices derived from HuD-Tg mice and WT littermates were subjected to immunofluorescent staining followed by image acquisition as described previously [8]. Fluorescent signals within the mossy fiber tracts were quantified using ImageJ (NIH) and the density of IF was calculated by normalization of the IF signal to the area of measurement.

## Results

### HuD selectively enhances expression of the BDNF long 3′UTR reporter through a highly conserved ARE

**Figure 1:**
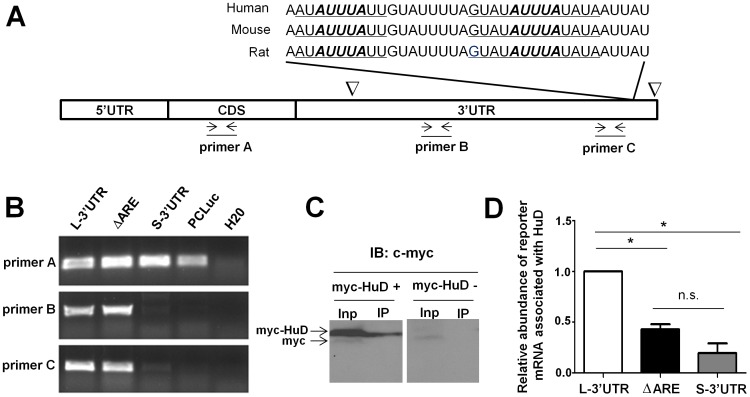
HuD enhances luciferase reporter expression through an ARE in the BDNF long 3′UTR. (A) A highly conserved cluster of Class I ARE in the BDNF long 3′UTR, with a consensus core sequence of *AUUUA* flanked by symmetric A or U (underlined). Triangles indicate alternative polyadenylation sites in the BDNF primary transcript. Primers used for RT-PCR to detect reporter mRNAs are illustrated underneath. (B) RT-PCR using multiple primers illustrated in (A) confirms expression of the expected RNA sequence from each transfected reporter construct. (C) Immunoblot (IB) showing expression of myc-HuD in the input (Inp) of transfected CAD cells and successful immunoprecipitation (IP) of myc-HuD. Two different anti-myc antibodies were used for IP and IB. (D) CLIP-qRT-PCR quantification of reporter mRNAs co-immunoprecipitated with HuD relative to the corresponding mRNA levels in the input. * indicates P<0.05 by Student's t-test (n = 3). Due to the presence of endogenous BDNF mRNA, primers specific for the luciferase coding region were used to detect reporter mRNAs. BDNF long 3′UTR reporter mRNA is preferentially enriched over short 3′UTR mRNA in immunoprecipitated HuD complex. Deletion of the ARE in the long 3′UTR significantly reduced the association or reporter mRNA with HuD.

AREs are prominent motifs found in mRNA 3′UTRs that recruit various ARE-binding proteins (ARE-BPs) to stabilize or destabilize target mRNAs [25]. AREs can be divided into three different classes: Class I AREs contain a core penta-nucleotide AUUUA flanked by A/U, Class II AREs contain overlapping AUUUA motifs, and Class III AREs do not contain typical AUUUA motifs but long stretches of U-rich sequences. Using the ARED-Organisms database (http://brp.kfshrc.edu.sa/ARED/), we identified a highly conserved Class I ARE specifically located in the BDNF long 3′UTR immediately up-stream of the distal polyadenylation site (Figure 1A), suggesting that ARE-BPs may preferentially regulate stability of the BDNF long 3′UTR mRNA.

HuD is a neuronal specific ARE-BP [26] that displays a functional spectrum substantially overlapping with that of BDNF [27]. To explore whether HuD interacts with the BDNF long 3′UTR via the ARE which in turn stabilizes the mRNA, we generated luciferase reporter constructs that carry the short BDNF 3′UTR (S-3′UTR), the full-length BDNF long 3′UTR (L-3′UTR) or the BDNF long 3′UTR lacking the ARE (ΔARE). The parental luciferase construct that does not harbor predictable ARE (PCLuc) and the aforementioned BDNF 3′UTR reporters were transfected individually into the immortalized brain neuronal cell line, CAD. Three sets of specific primers were employed in RT-PCR reactions to detect expression of the aforementioned reporter mRNAs, with primer A in the luciferase coding sequence (CDS), primer B located in the proximal portion of the long 3′UTR, and primer C located immediately up-stream of the ARE. As shown in Figure 1B, primer A detects comparable levels of all luciferase reporter mRNAs, whereas Primer B and C only detect the full-length BDNF long 3′UTR and the ΔARE reporter mRNA, confirming the expected expression of reporter mRNAs. Next, BDNF reporter constructs carrying L-3′UTR, ΔARE L-3′UTR and S-3′UTR were co-transfected with a c-myc-tagged HuD construct [28], followed by UV-cross linking immunoprecipitation (CLIP) using an anti-myc antibody and qRT-PCR. Successful immunoprecipitation of myc-HuD is shown in Figure 1C. Due to the presence of endogenous BDNF transcripts in CAD cells, primer A was used in this experiment to specifically address association of myc-HuD with the reporter mRNAs that carry various sequence segments in the BDNF 3′UTRs. As shown in Figure 1D, the long 3′UTR reporter mRNA is preferentially enriched in immunoprecipitated HuD complex compared to the short 3′UTR reporter mRNA. Furthermore, removing the ARE in the long 3′UTR significantly attenuated the association of the reporter mRNA with HuD.

**Figure 2:**
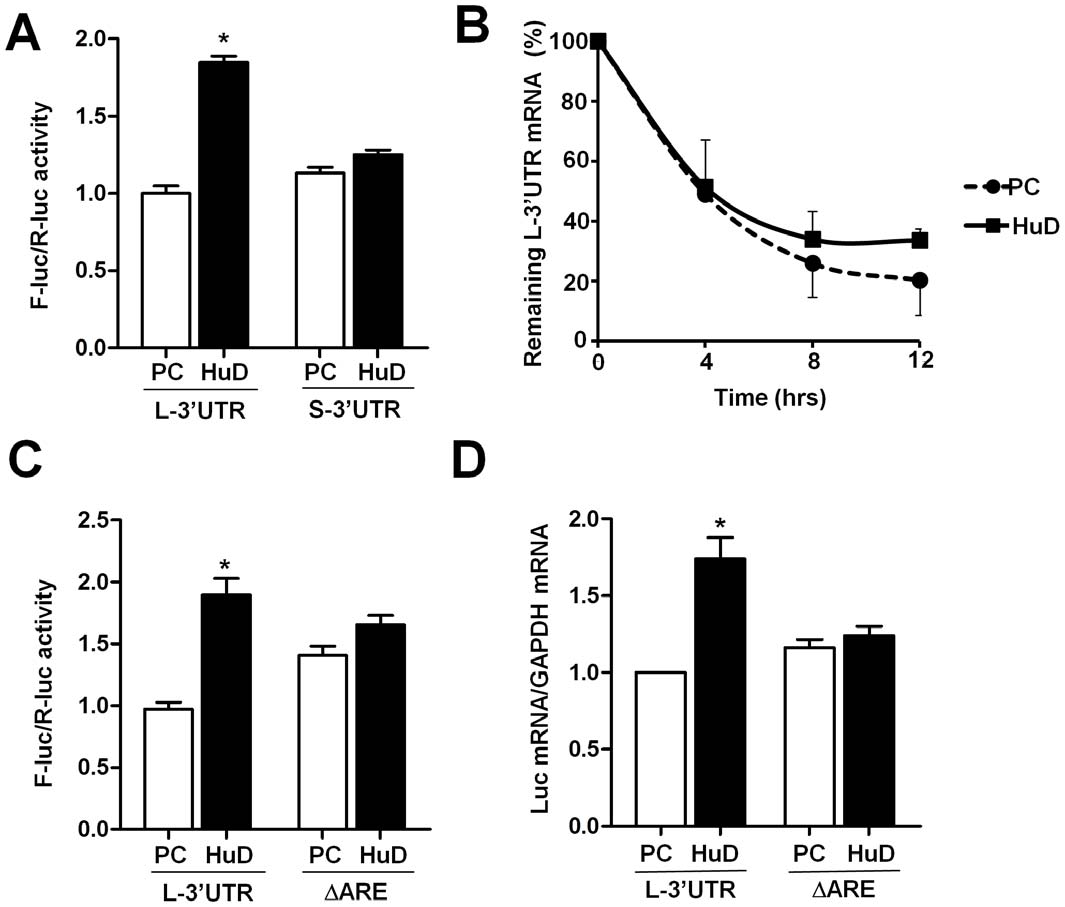
HuD selectively enhances expression of the luciferase reporter that harbors the BDNF long 3′UTR in an ARE-dependent manner. CAD cells were cotransfected by 200 ng of reporter construct together with either pcDNA-HuD (HuD) or the pcDNA parental vector (PC). 20 ng of pRL-CMV Renilla luciferase construct was included in each transfection. Twenty-four hours after transfection, cells were harvested and subjected to dual luciferase reporter assay or mRNA extraction followed by DNAase-treatment and RT-qPCR. (A) myc-HuD enhances expression of the luciferase reporter that carries the BDNF long 3′UTR but not the short 3′UTR. (B) myc-HuD reduces decay of the BDNF long 3′UTR reporter mRNA in co-transfected CAD cells in which transcription is inhibited by actinomycin D. (C) Loss of the ARE in the BDNF long 3′UTR abolished the repose to HuD-dependent enhancement of reporter expression. (D) HuD regulates reporter mRNA expression mediated by the ARE in the BDNF long 3′UTR. * indicates P<0.05 by Student's t-test (n = 3).

We next tested whether HuD can regulate reporter mRNA expression selectively via the BDNF long 3′UTR in an ARE-dependent manner. As shown in Figure 2A, myc-HuD significantly increased luciferase reporter expression from the full length BDNF long 3′UTR construct, but not the BDNF short 3′UTR construct. In addition, myc-HuD leads to a moderately reduced decay of the BDNFL-3′UTR reporter mRNA in the presence of the transcription inhibitor actinomycin D (Figure 2B). Furthermore, removing the ARE in BDNF long 3′UTR abolished the effect of HuD on luciferase reporter activity (Figure 2C). Importantly, the increase of BDNF long 3′UTR reporter mRNA by myc-HuD was also abolished when the ARE was removed (Figure 2D), recapitulating the HuD response measured by luciferase activity (Figure 2C). Thus, although HuD can also promote translation initiation [29], its regulation mediated by the BDNF long 3′UTR ARE most likely occurs at the level of mRNA stability.

### HuD directly binds and stabilizes RNA that carries the ARE segment in the BDNF long 3′UTR

**Figure 3:**
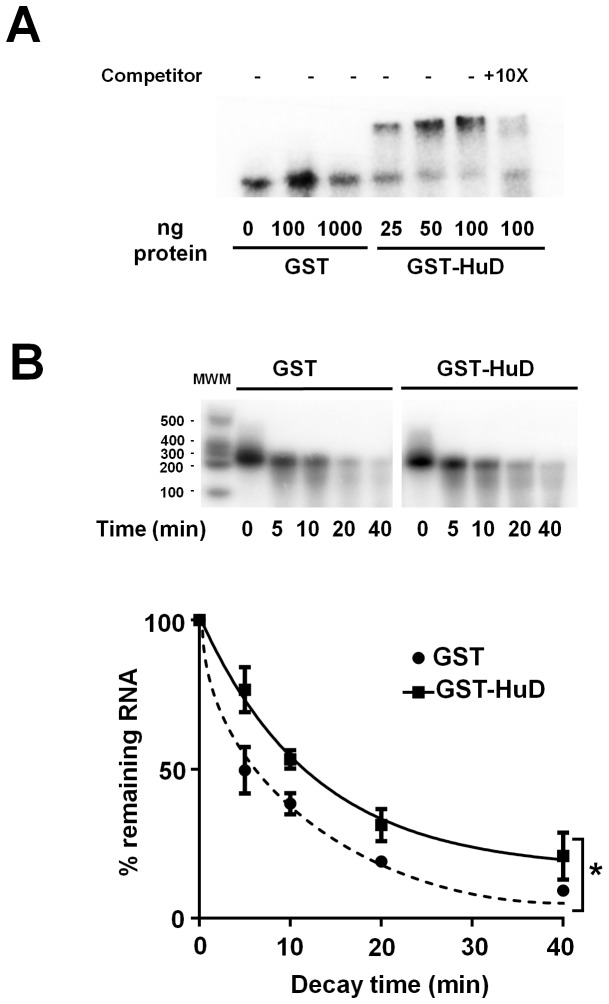
Binding of HuD to the ARE in the BDNF-long 3′UTR increases the stability of the mRNA. (A) Specific binding of HuD to the ARE in BDNF-long 3′UTR was demonstrated by REMSA using recombinant proteins and radiolabeled RNA along with a 10-fold molar excess of cold ARE competitor. (B) In vitro decay assay of capped and polyadenylated RNA containing the BDNF-ARE with left lane showing RNA molecular weight markers. Analysis of the rate of decay in three independent experiment revealed that the mRNA is stabilized in the presence of HuD. Half-life for GST-treated mRNA is 7.0±0.90 min, and for GST-HuD-treated mRNA is 12.0±1.0 min. ** indicates p<0.05 by Two way ANOVA with a quantitative factor (n = 3 separate experiments).

To test whether HuD can indeed directly bind the ARE segment in the BDNF long 3′UTR, we performed RNA-mobility shift assays using recombinant GST-HuD or GST, which is a commonly used approach for demonstrating direct interactions between an RNA-binding protein and the sequence element within its target mRNA. As shown in Figure 3A, GST-HuD bound the BDNF-ARE segment with high affinity. A mobility shift of the RNA can be clearly visualized with only 25 ng of GST-HuD in the presence of high concentrations of non-specific RNA competitor (tRNA, 0.25 mg/ml) and BSA (0.25 mg/ml). In contrast, GST alone did not bind the RNA at any concentrations examined. The specificity of HuD-ARE interaction was further demonstrated by the displacement of the radiolabeled RNA with a cold ARE competitor (Figure 3A, right panel).

Although myc-HuD increased the BDNF L-3′UTR mRNA in an ARE-dependent manner (Fig. 2D), this experiment could not exclude the possibility that HuD may also regulate other trans-acting factors which in turn stabilize BDNF L-3′UTR mRNA. To further examine whether interactions between HuD and the BDNF-ARE alone could result in RNA stabilization, we used *in vitro* transcribed capped and polyadenylated BDNF-long 3′UTR ARE RNA and GST-HuD for *in vitro* decay assays (Figure 3B). Addition of GST-HuD to brain extracts resulted in a significant stabilization of the RNA (Two way ANOVA, F = 4.68206. DFn = 1 DFd = 30, p = 0.03857), with an almost 2-fold reduction in the initial rate of decay. These data clearly indicate that HuD is capable of direct binding to the ARE in the BDNF long 3′UTR, which in turn leads to stabilization of the bound RNA.

### HuD expression levels selectively regulate the abundance of the endogenous BDNF long 3′UTR mRNA

**Figure 4:**
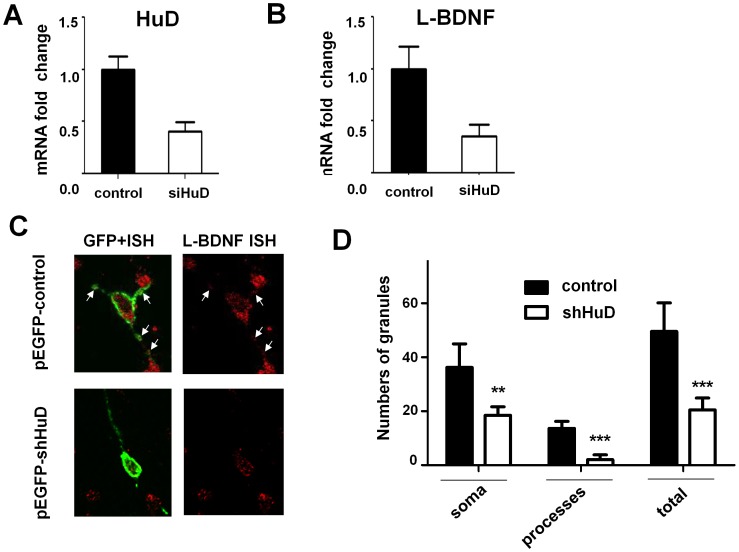
HuD knockdown reduced the levels of endogenous BDNF long 3′UTR mRNA. (A) siRNA knockdown of HuD in transfected CAD cells measured by qRT-PCR using a HuD-specific primer flanking the target site by the siRNA. (B) Reduction of endogenous BDNF long 3′UTR mRNA in CAD cells measured by qRT-PCR as a result of HuD knockdown. For (A) and (B), results were derived from 3 independent experiments (n = 3), * indicates P<0.05. (C) Representative confocal images of L-BDNF FISH (red) in E17 hippocampal neurons transfected with either pEGFP control vector or pEGFP-shHuD plasmid. The transfected cells was marked by the green fluorescence and the number of FISH grains were counted in the cell bodies and processes (n = 6 for each condition) and graphically displayed in (D). Arrows mark the FISH grains in the processes. Note that the number of ISH grains in the shHuD- treated cells decreased throughout the soma and neurite relative to control GFP-vector treated cells.

We next examined whether HuD abundance can govern the expression levels of endogenous long-BDNF mRNA. HuD was successfully knocked down in CAD cells using a previously published siRNA (Figure 4A), which leads to a significant reduction of the endogenous BDNF long 3′UTR mRNA (Figure 4B). We then performed shRNA-mediated HuD knockdown in primary cultured hippocampal neurons that express high levels of HuD and BDNF [22], [30]. Considering the dendritic localization of the BDNF long 3′UTR mRNA [9], we performed fluorescent in situ hybridization (FISH) using a BDNF long 3′UTR-specific probe and quantitatively measured the IF signals in the soma and dendrites of transfected neurons marked by co-expression of GFP. As shown in Figure 4C and D, shRNA-mediated HuD knockdown leads to significant reduction of long 3′UTR BDNF mRNA in both soma and dendrites, suggesting that HuD governs the level of the long 3′UTR BDNF mRNA in the entire neuron.

**Figure 5:**
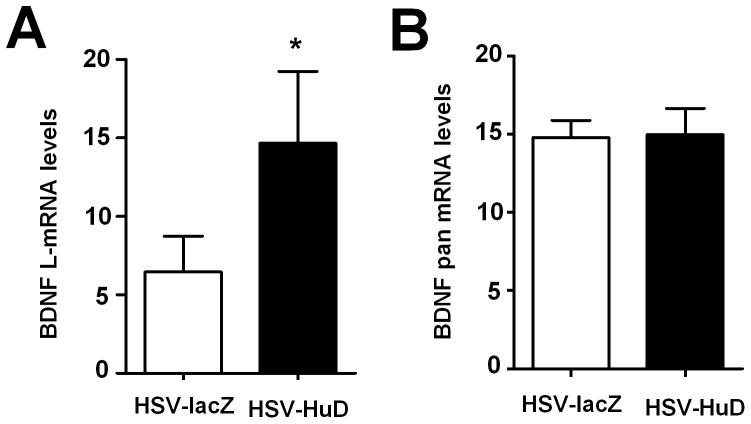
Forced expression of exogenous HuD selectively enhanced expression of BDNF long 3′UTR mRNA in primary cultured neurons. E17 cortical neurons were cultured for 2 days and then infected with HSV-HuD or HSV-lacZ virus. Following 3 days in culture, the levels of long 3′UTR BDNF mRNA and pan BDNF mRNA were determined by qRT-PCR using primers specific in the long 3′UTR (A) and primers in the coding region that detects the pan BDNF mRNA (B). *p<0.05 (Student's t-test, n = 4).

Reciprocally, we treated cortical neurons with HSV vectors to achieve forced expression of exogenous HuD [22]. The control virus that expresses the LacZ gene was used to treat parallel cultures. As shown in Figure 5A, over-expression of HuD significantly increased the levels of the BDNF long 3′ UTR mRNA, whereas the pan BDNF mRNA levels were unaltered (Figure 5 B), in which the short BDNF mRNA is the major isoform [8]. Together, these results suggest that HuD abundance indeed governs neuronal BDNF long mRNA expression.

### A HuD transgene selectively up-regulates BDNF long 3′UTR mRNA and BDNF protein in the hippocampal MF pathway

**Figure 6:**
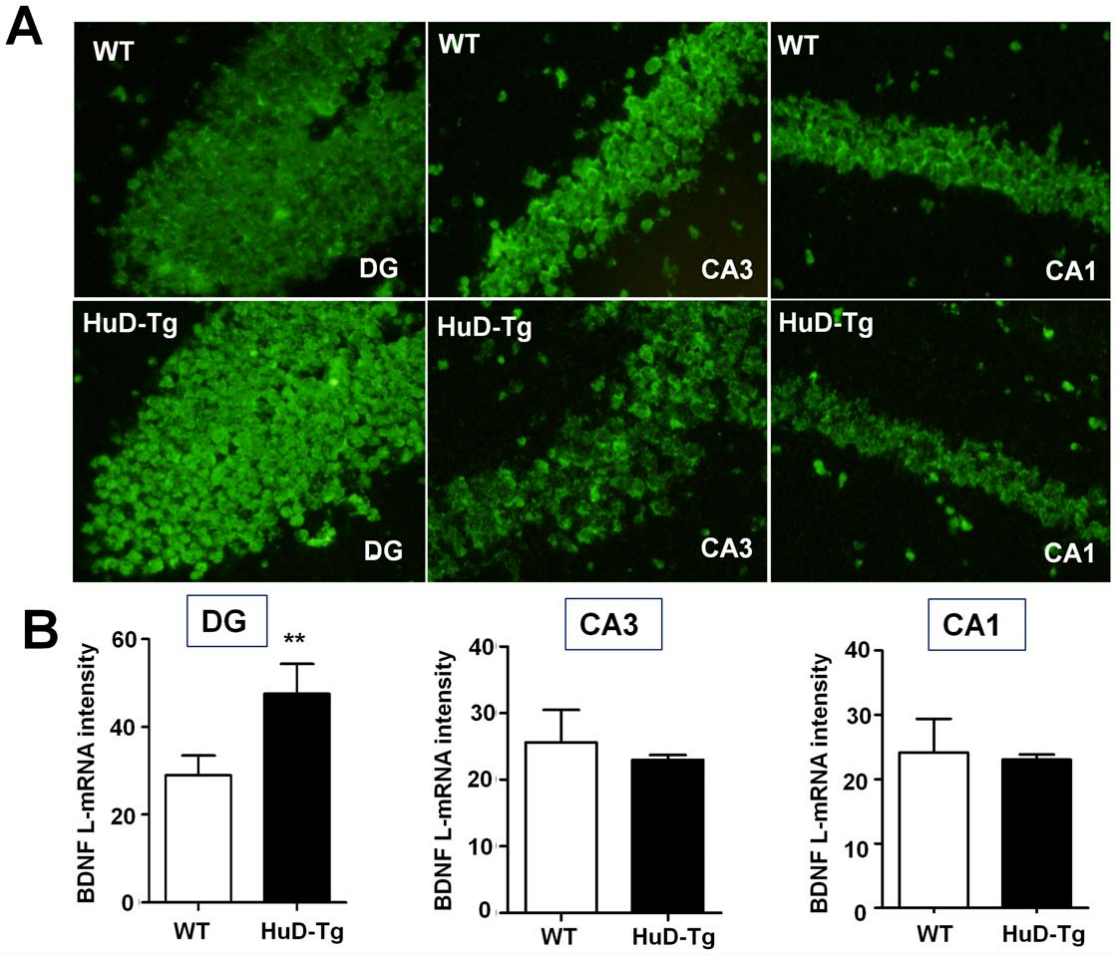
A HuD transgene enhances expression of the BDNF long 3′UTR mRNA in hippocampal DGCs. (A) Representative FISH images of fluorescent in situ hybridization of BDNF long 3′UTR mRNA in the neuronal soma layers of hippocampal DG, CA3 and CA1 of HuD-Tg and WT littermates (scale bar = 75 µm). (B) Quantification of FISH intensity in four pairs of HuD-Tg and WT controls. ** p<0.01 (Student's t-test, n = 4).

Finally, we tested whether elevated HuD expression can regulate neuronal BDNF production *in vivo* through the long 3′UTR. Quantitative FISH was performed using brain slices of HuD transgenic mice (HuD-Tg) that express myc-tagged HuD under the CamKIIα promoter [31]. Non-transgenic WT littermates were processed in parallel as baseline controls. Consistent with the preferential increase of HuD from the transgene in HuD-Tg hippocampal DGCs [31], BDNF long 3′UTR mRNA is significantly up-regulated in the DGCs of HuD-Tg (Figure 6A and B). In contrast, BDNF long 3′ UTR mRNA was not increased in CA3 or CA1 neurons (Figure 6), conceivably explained by the fact that the HuD transgene is not significantly over-expressed in CA3 and CA1 pyramidal neurons [31].

**Figure 7:**
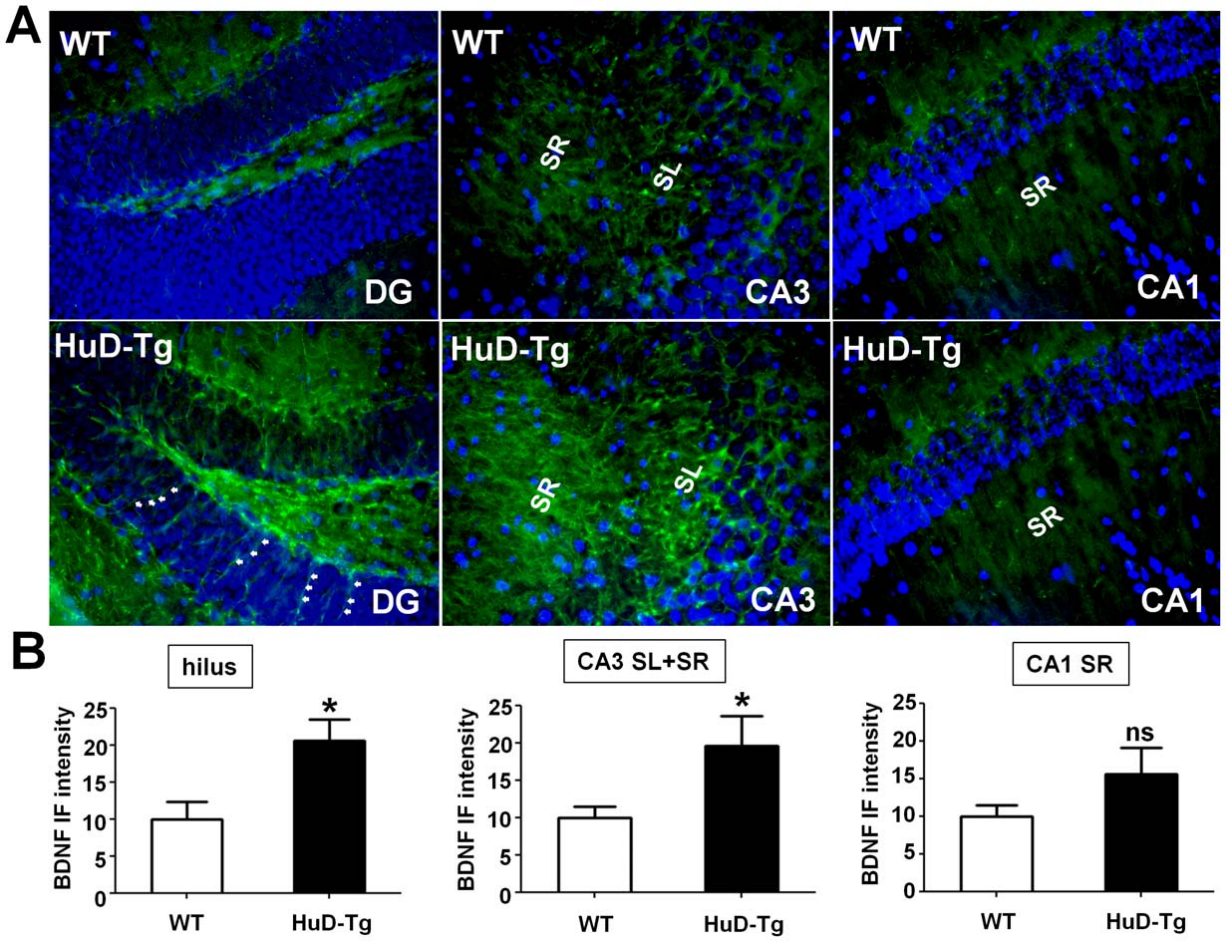
The HuD transgene enhances BDNF protein levels in the hippocampal MF pathway. (A) Representative images of BDNF immunofluorescence (green) in the hippocampal hilus, CA3 and CA1 regions in HuD-Tg and WT littermates. DAPI stained nuclei (blue) mark the aforementioned principle neuron layers. Asterisks indicate enhanced BDNF signals in the long processes of adult neural stem cells in the SGZ. (B) Quantification of BDNF immunofluorescence in the hilus, CA3 strata lucidum (SL) and strata radiatum (SR) and CA1 stratum radiatum (SR) are graphically displayed. *p<0.05 (Student's t-test, n = 3).

BDNF protein synthesized in DGCs is primarily transported to and stored in hippocampal MF axons that project through the hilus to form large synapses with CA3 dendrites [32]. Consistent with the enhanced expression of BDNF long 3′UTR mRNA in DGCs (Figure 6), significantly increased BDNF protein immunofluorescence was detected in the hilus and the CA3 strata lucidum and radiatum of HuD-Tg as compared to the WT control (Figure 7A and B). Transgenic HuD expression also increased BDNF protein in cells of the DG subgranular zone (SGZ) that is enriched of adult neural stem cells [33], particularly in the characteristic long processes extended into the DGC layer (arrows in Figure 7A). In contrast, BDNF protein levels in HuD-Tg CA1 neurons are not significantly altered (Figure 7A and B). Given the vigorous regulation of endogenous HuD in DGCs [20], HuD-dependent stabilization of BDNF long 3′UTR mRNA is a novel mechanism that controls BDNF production in the hippocampal MF pathway, a critical circuitry that governs hippocampal excitatory activities in physiological and pathological plasticity during epileptogenesis [32], [34].

## Discussion

Our studies identify HuD as the first RBP that selectively binds to and stabilizes the BDNF long 3′UTR mRNA but not the short BDNF mRNA. The interaction and function of HuD is mediated by an ARE in the distal end of BDNF long 3′UTR, which is highly conserved in mammalian species. Furthermore, we provide evidence that the cellular abundance of HuD in neurons preferentially regulates the expression levels of the BDNF long 3′UTR mRNA isoform in culture and *in vivo*. Considering the vigorous regulation of HuD and BDNF during neuronal differentiation and neuronal activation [2], [35], [36], the functional connection between HuD and BDNF uncovered by our studies provides a novel mechanism that may synergistically govern normal brain development and accommodate functional changes in the brain.

In a recent report, HuD was shown to associate with several neurotrophic factor mRNAs, including BDNF [30]. In addition, manipulating HuD expression correlated with altered expression of these neurotrophic factor mRNAs. However, whether the long or the short BDNF 3′UTR interacts with HuD was undetermined. Whether HuD controls stability of BDNF mRNA and whether a specific ARE in BDNF 3′UTR is required for HuD to regulate BDNF is unknown. Moreover, whether HuD abundance can regulate BDNF mRNA in vivo remains elusive. These are important questions given the two distinct BDNF 3′UTRs differentially regulate BDNF translation and dendritic localization in response to neuronal activation [8], [9]. In this study, we demonstrated that a highly conserved ARE in the distal end of the BDNF long 3′UTR primarily mediates the association of the reporter mRNA with HuD in transfected cells and directly interacts with recombinant HuD in vitro with high specificity and affinity. We also show that recombinant HuD can stabilize an mRNA that contains the ARE in the BDNF long 3′UTR in brain lysates. Furthermore, HuD preferentially regulates the endogenous BDNF long 3′UTR mRNA in both the somatic and dendritic compartments of brain neurons. In contrast, the short BDNF3′UTR reporter mRNA does not appear to be regulated by HuD. Noticeably, mRNAs carrying AREs in the 3′UTR often display short half-lives [25], [37]. Consistent with his view, the BDNF long 3′UTR mRNA is less stable than the BDNF short 3′UTR mRNA [10], likely due to recruitment of undefined destabilizing RBPs and/or miRNAs by the unique sequence in the long 3′UTR. Such instability provides a practical opportunity for stabilizing RBPs, represented by HuD, to selectively increase the abundance of BDNF long 3′UTR mRNA. This is of particular interest, considering the fact that the long 3′UTR, but not the short 3′UTR, mediates activity-dependent translation of BDNF in brain neurons [8].

Despite its primary localization in the neuronal soma, the BDNF long 3′UTR mRNA is also delivered to dendrites, which is critical for controlling dendritic BDNF protein levels and synaptic maturation [9]. Thus, molecular mechanisms governing dendritic BDNF mRNA abundance have attracted enormous attention. Interestingly, HuD is also detected in dendrites, although the majority of HuD is present in the neuronal soma [30], [36], [38]. We show that shRNA-mediated HuD knockdown reduces the long BDNF mRNA isoform in both the somatic and dendritic compartments of hippocampal neurons in culture, suggesting that HuD plays essential roles to govern the stability of the BDNF long 3′UTR mRNA in both compartments.

Importantly, HuD expression is vigorously regulated during neuronal development, nerve regeneration and functional changes of the brain [39]. HuD protein is highly expressed in the embryonic and neonatal brain, but reduced to low levels in the adult brain where it is subjected to marked up-regulation upon neuronal activation in multiple plasticity paradigms [20], [27], [36], [38], [40], [41]. We found that over-expression of HuD in primary cultured cortical neurons selectively increases the BDNF long 3′UTR mRNA, but not the pan BDNF mRNA. Thus, the previously reported function of HuD in up-regulating BDNF must be specifically mediated by the BDNF long 3′UTR. It is important to point out that HuD is differentially regulated in distinct neuronal subpopulations in the brain in various functional paradigms [27], [42]. Along with the distinct expression patterns of the short and long BDNF mRNA isoforms in various brain regions [9], HuD-dependent selective stabilization of the BDNF long 3′UTR provides a novel posttranscriptional mechanism that increases the complexity of temporal and spatial regulation of BDNF to accommodate brain development and function.

A particularly interesting case is the regulation of HuD in hippocampus. HuD protein is not normally detected in adult hippocampal DGCs [20]. However, upon seizure induction, HuD is drastically up-regulated in DGCs. In contrast, although HuD is present in CA1 and CA3 neurons at rest, it is only moderately increased after seizures. Recapitulating the robust increase of HuD in DGCs in response to seizure, resting HuD-Tg mice display the highest fold increase of HuD protein in DGCs [24], [31], although HuD transgene expression can be detected in all forebrain neurons. The relatively lower level of over-expression of the HuD transgene in CA1 explains the lack of significant increase of BDNF protein in these neurons. In contrast, BDNF protein expression is preferentially increased in DGCs, which is transported and stored in the axonal terminals of the MFs. Considering the well-known effects of BDNF in promoting the growth of neuronal processes [34], increased BDNF expression in hippocampal MFs is likely a contributing factor for the MF over-projection in HuD-Tg [24]. Conceivably, the seizure-induced HuD up-regulation in DGCs could contribute to the well-characterized preferential increase of BDNF in the MF terminals and MF sprouting during epileptogenesis [32], [43].

Taken together, our study reveals a novel mechanism that controls BDNF expression through HuD-dependent stabilization of the BDNF long 3′UTR mRNA, which would impact various neuronal populations that harbor differential regulation of HuD in response to neuronal activity changes. However, because conventional knockout of HuD results in abnormal neuronal development [44], definitive demonstration of the biological consequence of HuD-dependent BDNF mRNA stabilization through the long 3′UTR is a challenging question that may only be addressed once inducible knockout of HuD can be achieved. Besides controlling mRNA stability, HuD was recently reported to enhance translation initiation through unwinding structural 5′UTRs [29]. Noticeably, the long 3′UTR BDNF mRNA is repressed for translation initiation in resting neurons but drastically activated upon neuronal and synaptic stimulation [8]. Thus, although the ARE in BDNF long 3′UTR appears to mediate HuD's effect primarily through mRNA stability in resting neurons, whether HuD can also enhance BDNF translation upon neuronal activation, perhaps involving interaction between the long 3′UTR and distinct 5′UTRs in BDNF mRNA isoforms, is an intriguing question to be answered by future studies.

### Ethics Statement

This study was carried out in strict accordance with the recommendations in the Guide for the Care and Use of Laboratory Animals of the National Institutes of Health. The protocol was approved by the Animal Care and Use Committee at Emory University (Protocol Number: 2000404).
